# Predictors of response after single session interventions for emotional distress: using enhanced psychoeducation in crisis situations

**DOI:** 10.1192/j.eurpsy.2025.1995

**Published:** 2025-08-26

**Authors:** A. L. D. S. Ache, B. B. Montezano, B. P. Mosqueiro, M. A. Caldieraro, L. Spanemberg, G. A. Salum, M. P. Fleck

**Affiliations:** 1 Psychiatry, Hospital de Clinicas de Porto Alegre; 2 Psychiatry, Programa de Pos-Graduacao em Psiquiatria e Ciencias do Comportamento; 3 Escola de Medicina, Programa de Pos-Graduacao em Medicina e Ciencias da Saude, Escola de Medicina, Pontifıcia Universidade Catolica do Rio Grande do Sul; 4 Escola de Direito, Programa de Pos-Graduacao em Ciencias Criminais, Escola de Direito, Pontifcia Universidade Catolica do Rio Grande do Sul, Porto Alegre, Brazil

## Abstract

**Introduction:**

The COVID-19 pandemic had a major impact on the world’s emotional health, and knowledge is limited about the efficacy of traditional interventions in this context. The TelePSI Project, initiated by the Hospital de Clınicas de Porto Alegre in collaboration with the Brazilian Ministry of Health, aimed to provide online

mental health care to essential services professionals during the pandemic. Single session of enhanced psychoeducation (EP) is an innovative strategy proposed by TelePSI, suggesting lifestyle changes based on individual risk and protective factors or physical, emotional, psychological, and well-being.

**Objectives:**

Single-session interventions are an effective strategy for reducing emotional distress. Enhanced psychoeducation, which includes empathic listening, risk stratification, symptom monitoring, and habit modification is particularly suitable for single-session interventions. We investigated predictors of response to an online enhanced psychoeducation intervention among essential service professionals during the pandemic in Brazil.

**Methods:**

The TelePSI Project, financed by the Brazilian Ministry of Health, was a nationwide initiative that served more than 3,300 individuals in various psychotherapeutic modalities. Data were collected from April 2020 to December 2021. We included all participants with high levels of emotional distress who received single-session interventions

**Results:**

Final sample 460 individuals (89.1% women, 81.1% health professionals). After 1 month, 300 participants were reassessed. Overuse of social media, use of social networks to contact family and friends, playing video games, smoking, drinking alcohol, and spending time with pets were associated with less improvement in symptoms, whereas playing an instrument and previous psychological treatment were associated with greater symptom improvement. This highlights the impact of lifestyle factors on the efficacy of single-session interventions.

**Image:**

**Figure:**
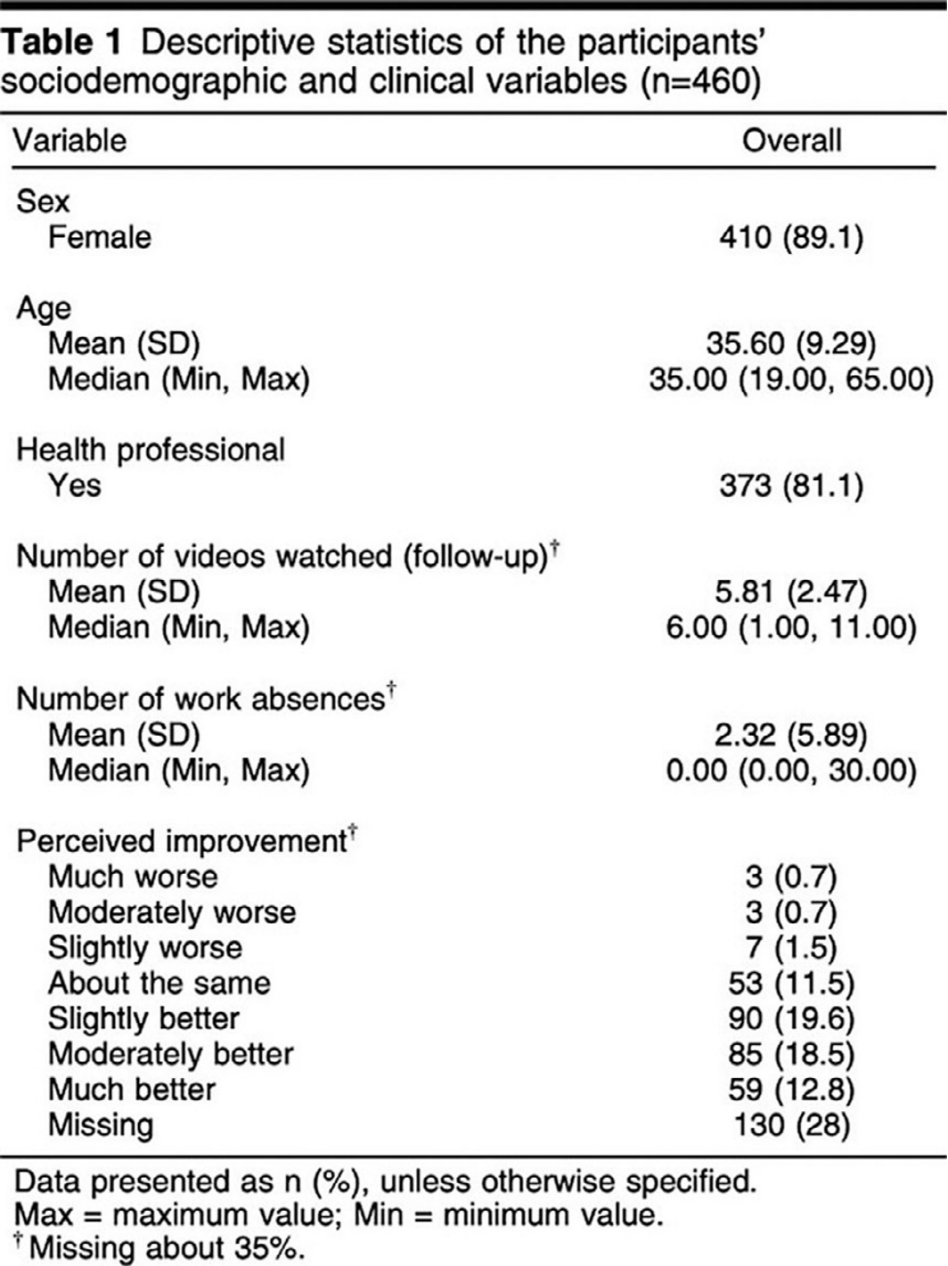

**Image 2:**

**Figure:**
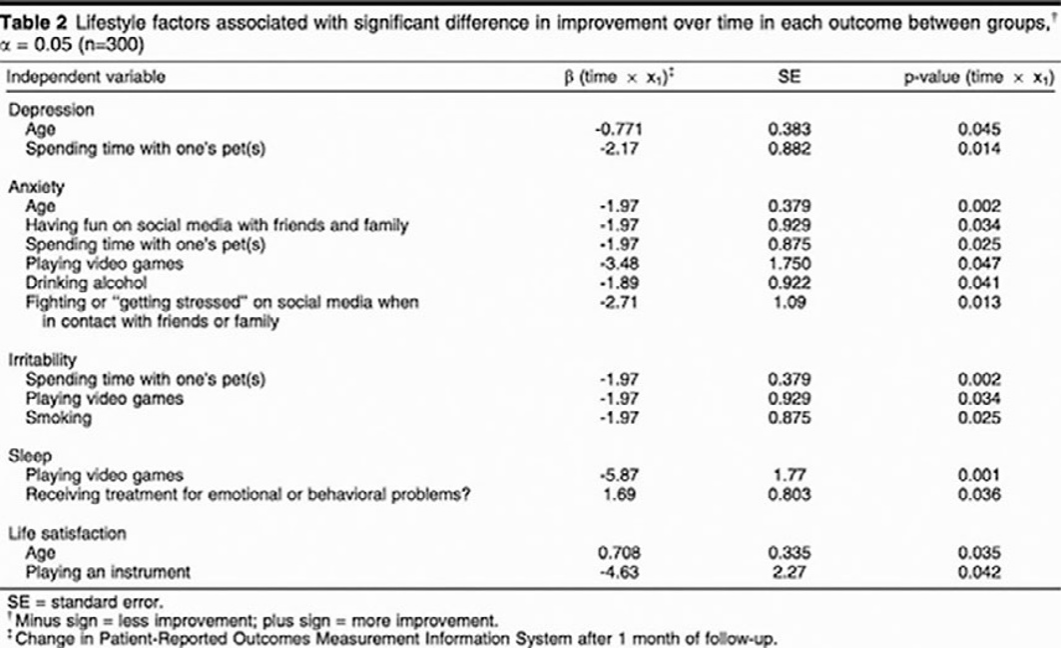

**Image 3:**

**Figure:**
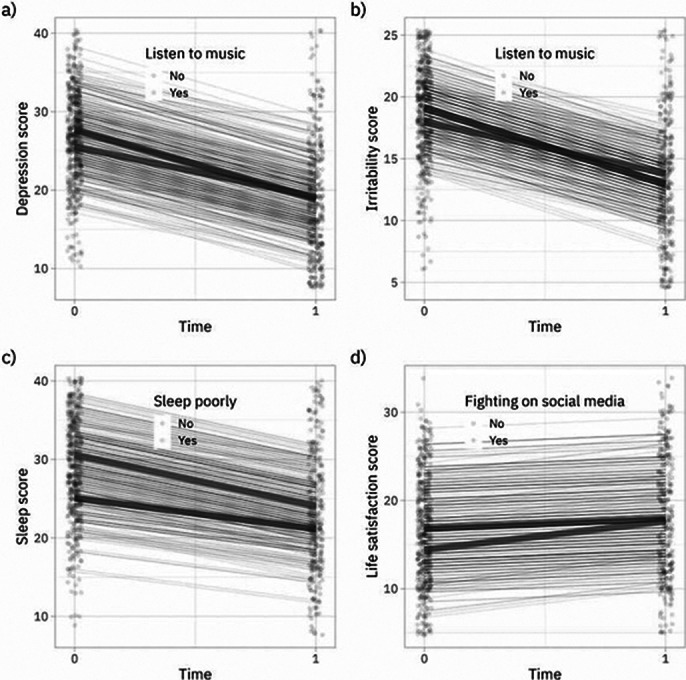

**Conclusions:**

Online EP with support videos, as proposed by TelePSI, appears to be an effective intervention strategy for healthcare professionals with symptoms of anxiety, depression, and irritability. Although a significant proporion of the participants improved, some variables were associated with less improvement, such as spending time with pets, tobacco use, alcohol use, and playing video games. In this study, we set out to analyze variables associated with participant improvement because, by evaluating these factors, we can better understand this new model of psychoeducation, which represents a promising alternative for individuals during crises such as the COVID-19 pandemic. Furthermore, the intervention should be explored beyond the context of the pandemic and social isolation. Further research on TelePSI is forthcoming, including a comparison of intervention arms

**Disclosure of Interest:**

None Declared

